# Sleep Disturbance Scale for Children: Italian Validation in Autism Spectrum Disorder Population

**DOI:** 10.3390/ijerph191610163

**Published:** 2022-08-16

**Authors:** Ester Mignolli, Alessia Scialpi, Donatella Valente, Anna Berardi, Giovanni Galeoto, Marco Tofani

**Affiliations:** 1 Department of Anatomical, Histological, Forensic and Orthopaedic Sciences, Sapienza University of Rome, 00185 Rome, Italy; 2 Department of Human Neurosciences, Sapienza University of Rome, 00185 Rome, Italy; 3 Neuromed—Istituto Neurologico Mediterraneo, Istituto di Ricovero e Cura a Carattere Scientifico—IRCCS, 86077 Pozzilli, Italy; 4 Professional Development, Continuous Education and Research Service, Bambino Gesù Children’s Hospital, Istituto di Ricovero e Cura a Carattere Scientifico—IRCCS, 00165 Rome, Italy

**Keywords:** sleep, sleep disorder, autism spectrum disorder, insomnia, children, parents, quality of sleep, outcome measure, validation

## Abstract

Sleep disorders in children with autism spectrum disorders (ASDs) are well-described. However, there is a lack of specific assessment tools to investigate sleep disturbance in this target population. The present investigation reports the Italian validation of the Sleep Disturbance Scale for Children (SDSC) in the ASD population, also investigating the correlation between sleep disorders In both children and parents. Internal consistency and test–retest reliability were investigated using Cronbach’s alpha and intraclass correlation coefficient (ICC), respectively. Concurrent validity was analyzed by comparing the score of the SDSC with the Pittsburgh Sleep Quality Index (PSQI), while the correlation between the SDCS score and the General Sleep Disturbance Scale (GSDS) was used to analyze the correlation between sleep disorders in children and sleep disorders in their parents. In total, 99 children with a diagnosis of ASD participated in the study. Cronbach’s alpha revealed satisfactory value (0.853), as well as reliability (ICC 0.972) and concurrent validity (0.745). Our results also revealed a significant linear correlation between children’s and parents’ sleep disorders (*p* < 0.05). In conclusion, we found the SDSC to be a useful tool for measuring sleep disorders in ASD children. Our findings offer concrete inputs to achieve adequate pathways for taking care of children with ASDs and their parents.

## 1. Introduction

Sleep is a physiological process in which the reduction in consciousness and metabolism occurs; it affects mental and physical health and is essential for the normal functioning of all human body systems, including the immune system [1]. Sleep quality is a vital indicator of overall health and well-being. The National Sleep Foundation revisited the guidelines’ criteria on sleep needs for each age group [2]: the recommended amount of sleep for school-age children (6–13 years) was extended by one hour to 9–11 h, and the amount of sleep for adolescents (14–17 years) was extended by one hour to 8–10 h.

Insomnia is one of the most common health problems in clinical practice, and among all sleep disorders, it is certainly the most prevalent, as it is estimated to occasionally affect more than half the population and afflicts at least 9 million Italians [3]. It is associated with significant functional impairment, high health care costs, and various medical comorbidities [4]. However, poor sleep quality can have negative repercussions in children with neurodevelopmental disabilities, as well as in their participation in all activities of daily life [5]. Some authors have highlighted that about 35–46% of children suffer from some sleep disorders, and insomnia represents the main prevalent disorder [5,6]. According to the International Classification of Sleep Disorder and the Diagnostic and Statistical Manual Mental Disorders 5 (DSM-V) [7], the diagnosis of insomnia is primarily based on the assessment of self-reported symptoms, and the sleep diary has become a standard tool for assessing the patient’s perception of self-reported insomnia.

The use of structured and semi-structured clinical interviews to assess the nature, history, and severity of sleep difficulties is increasing in clinical practice [8]. The Children’s Sleep Habits Questionnaire (CSHQ) [9] is a parent-reported questionnaire consisting of 45 items investigating the frequency of sleep disorders in children during the previous week. The Paediatric Sleep Questionnaire (PSQ) [10] is divided into 10 sections with a total of 22 items with a dichotomic answer (yes or no); it can be easily filled out by parents of children between 2 and 18 years and by young people. The Sleep Disturbance Scale for Children (SDSC) [11] is a tool for the assessment of sleep disorder frequency over six months prior to administration. The main strengths of the SDSC are the simplicity of questions and the resulting immediacy in measuring sleep quality. These features make the SDSC an ideal tool in clinical settings and also for epidemiological studies.

Autism spectrum disorder (ASD) is a condition characterized by abnormalities in behavior, deficits in communication, language, and social interactions; restricted and repetitive interests are also reported [7]. Sleep disorders in ASDs are noted [12,13], and different studies continue to find parent-reported sleep problem rates ranging from around 50% to 80% for children with an ASD, compared with 9% to 50% for comparison groups [14,15]. The sleep disorders most frequently observed in these children [16] include delay in sleep onset, frequent nocturnal awakenings, and reduced sleep duration. Furthermore, in children with ASDs who have sleep disorders, worsening behavioral problems (mood instability, aggression, self-injury) are observed throughout the day, making children’s management even more difficult for caregivers [17]. Sleep disorders may adversely affect children’s daily function, behavior, learning, memory regulation, and cognition [18]. It may also cause emotional problems such as aggression, irritability, over-reactivity, and depression [19]. Moreover, disorders also negatively impact ASD symptoms [20]. For example, ASD children with sleep problems showed more severe social skills deficits, and they scored lower on social tests [21].

Considering there is no validation of a specific tool for the ASD population in Italy, our primary objective was to analyze the psychometric properties of the SDSC in a population of children with a diagnosis of ASD. As a secondary objective, we also explored the relationship between the sleep quality of children with ASDs and that of their parents.

## 2. Materials and Methods

The present study was conducted by a research group from the Sapienza University of Rome together with the Rehabilitation and Outcome Measures Assessment (R.O.M.A.) Association. In the last few years, this research group has dealt with different studies related to ASDs and the validation of different outcome measures [22,23,24,25,26,27,28,29].

### 2.1. Sampling and Procedures

Parents or caregivers of children with a diagnosis of ASD between 3 and 16 years old were recruited for the study throughout different health centers in Italy, namely Istituto Leonarda Vaccari of Rome, Casa di Cura INI- Villa Dante in Guidonia, Anffas in Rome, the Logos Center in Taranto, and within the Department of Human Neurosciences at the Sapienza University of Rome. Diagnoses of both ASD and insomnia were evaluated by medical doctors specialized in child neurology and psychiatry according to the International Classification of Sleep Disorder and the DSM-V [30]. Children who take medications or have other comorbidities were excluded. All parents were informed about the study purpose, procedures, and timing for administering the tool. Informed written consent was obtained for each child whose parents agreed to participate.

### 2.2. Assessment Tools

Sleep Disturbance Scale for Children (SDSC) [11] is a rating scale that investigates the frequency of sleep disturbances in the previous six months in children aged 3–16 years. It is a parent-reported assessment tool consisting of 26 items with a 5-point Likert scale ranging from 1 (never) to 5 (always). The SDCS is divided into six subscales: DIMS = disorders initiating and maintaining sleep (7 items); SBD = sleep breathing disorders (3 items); DA = disorders of arousal (3 items); SWTD = sleep–wake transition disorders (6 items); DOES = disorders of excessive somnolence (5 items); and SHY = sleep hyperhidrosis (2 items). The total score is calculated by summing the scores of each subscale ranging from 26 to 130.

The Pittsburgh Sleep Quality Index (PSQI) [31,32] is a 19-item self-assessment questionnaire measuring sleep quality in the previous month. The questionnaire assesses seven domains: sleep quality, sleep latency, sleep duration, habitual sleep efficiency, sleep disturbances, use of sleeping medications, and daytime dysfunction. These domains are rated on a scale ranging from 0 to 3.

The General Sleep Disturbance Scale (GSDS-IT) [33] is a self-report (Likert-type) scale that assesses how often sleep-related experiences occurred in the previous week. It consists of 21 items divided into 6 subscales: initial insomnia (1 item), maintenance insomnia (2 items), sleep quality (8 items), sleep quantity (2 items), daytime function (6 items), and use of sleep-inducing substances (6 items). The score for each question is rated using an 8-point scale ranging from 0 (no days) to 7 (all days). The total scale score is the sum of the scores for each item ranging from 0 to 147.

### 2.3. Data Analysis

All statistical analyses were performed with the Statistical Package for Social Science (SPSS) software. Internal consistency was examined with Cronbach’s α to assess the interrelatedness of the items and the homogeneity of the scale. A value of α ≥ 0.7 is commonly considered an acceptable indicator [34]. To assess the test–retest reliability, a randomized sample of the entire population underwent a second administration 25 days after the first evaluation. To determine the degree to which repeated measurements are free from measurement error, the intraclass correlation coefficient (ICC) was calculated. A value of 0.70 or greater is considered satisfactory [35]. To assess concurrent validity, the Italian version of the PSQI was used because it was already validated in the target population [36,37]. The PSQI was administered to the entire population and scores were compared with those of the SDSC using Pearson’s correlation coefficients. Pearson’s correlation coefficients ranged from 0 (indicating no linear relationship between variables) to +/−1 (indicating a perfect linear relationship), and values were interpreted as follows: <+/−0.3 indicate a weak relationship, those between +/−0.3 and +/−0.69 indicate a moderate relationship, and values ≥+/−0.7 indicate a strong relationship. The same coefficient was used to investigate the correlation between the sleep quality of children with ASDs and that of their parents, confronting the score of the GSDS and SDSC.

## 3. Results

For the present study, 102 children with diagnoses of ASD were recruited from different rehabilitation centers. Of these, 99 agreed to participate and were included in the study. The demographic characteristics of the population are summarized in Table 1.

The SDSC demonstrated good internal consistency, with a Cronbach’s α of 0.853. Table 2 reports the item–total correlations, revealing that all components concurred with the structure of good internal consistency.

The SDSC showed good test–retest reliability, with an ICC of 0.972 for the total score, ranging from 0.916 and 0.991. Values for each SDSC subscale are reported in Table 3.

**Figure 1:**
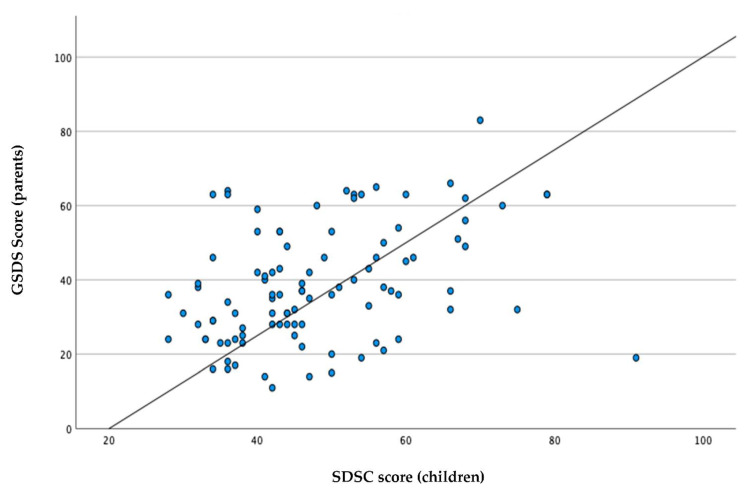
Correlation between sleep disorders in children and their parents.

The Italian version of the PSQI was administered to the whole population. Pearson’s correlation coefficient showed statistically significant values and a positive correlation between the subscales and the total scores, indicating a very good correlation between the total score of the SDSC and that of the PSQI, with a value of 0.745 and a *p* < 0.01. The results are summarized in Table 4. The correlation analysis between the GSDS and the SDSC, as measured by Pearson’s correlation coefficient, showed statistically significant values and a weak linear correlation between subscales. Table 5 summarizes these findings. We also conducted a scatter plot analysis showing the existence of a linear correlation between the SDSC scale and the GSDS scales (Figure 1).

## 4. Discussion

In the present study, the first specific validation of the SDSC for children with ASDs was reported, which revealed good internal consistency, reliability, and concurrent validity.

Measuring sleep disorders in ASD patients is a compelling challenge. Although sleep disturbances are not inherent to the ASD condition, they are clearly more prevalent in children with ASDs than in typically developed children [38]. Sleep disorders in ASD children seem not to be related to age or IQ [39], but there is extensive evidence indicating that inadequate sleep can negatively impact cognitive, emotional, and behavioral functioning [40]; therefore, screening and assessing for sleep disturbances in children with ASDs is critical for their care [41,42].

At the outset of our investigation, we intended to validate the SDSC for ASD children. With regard to internal consistency, as measured with Cronbach’s alpha, our results are in line with those of the original [11], French [43], Turkish [44], Chinese [45], and Iranian [46] validations and higher than those of the Brazilian–Portuguese reports [47]. This finding confirms the good internal consistency of the scale and relatedness of the items. For test–retest reliability, we found a good value for the total score (0.927), in line with the Chinese version [45] and higher than the original [11]. This means that the SDSC has great stability over time and confirms its use for clinical practice. Furthermore, the SDSC showed a good Pearson’s correlation coefficient with the PSQI, demonstrating good concurrent validity. Concurrent validity measures how well a new test (SDSC) compares to a similar and well-established test (PSQI) that can measure the same construct.

Interesting topics emerge from the analysis of the correlation between sleep disorders in both children and their parents. Our results revealed a linear positive correlation between sleep disorders in parents, as measured with the GSDS, and sleep disorders in children with ASDs, as measured with the SDSC. In fact, when sleep problems are present in children with ASDs, they have a greater effect on parents’ sleep than when similar sleep problems are present in typically developing children [48]. The caregivers of children with ASDs are noted to have adverse psychological states that manifest as stress, depression, and anxiety [49]; mothers have more parenting stress than fathers, and their stress seems not to have a significant correlation with the severity of symptoms [50]. One study found that over two-thirds of parents of children with autism or Asperger’s disorder reported their own sleep was disrupted due to their child’s sleep [14]. Furthermore, compared with parents of typically developing children, parents of children with ASDs reported poorer sleep quality as well as an earlier morning wake time and shorter sleep duration [51]. Some investigators have suggested that after dealing with a child’s challenging behaviors during the day, disrupted sleep patterns may be reported as more severe by parents of children with ASDs [52]. Efforts should be made to address some of the unmet psychosocial needs of caregivers of children with ASDs.

### Limitations

Despite these encouraging results, our study has some limitations that need to be addressed. We did not investigate the differences in sleep disorders in parents across gender. The sample was likely limited, but further research should investigate the different manifestations of sleep disorders between mothers and fathers. Second, we did not use objective measures for detecting sleep disorders, but diagnoses were performed from a clinical perspective. However, it is also worth noting that polysomnography and/or actigraphy should be considered to provide more precise and accurate estimates of sleep disturbances and to reduce measurement errors related to these problems. These objective measures can also allow researchers to estimate the discriminant validity of the SDSC in the target population. Third, we did not investigate differences in sleep disorders across different age groups; however, sleep quality, as well as sleep disturbance manifestations, can differ between children and adolescents. Thus, further studies should investigate these aspects and verify the cross-cultural validity of the SDSC for the target population.

## 5. Conclusions

The present research revealed the SDSC as a valid and reliable tool for measuring sleep disturbance in children with ASDs. As a result, healthcare professionals can use it more often, and with more confidence, to better understand the sleep-related problems of the target population. This can help clinicians to improve the quality of care in both clinical and community settings, also supporting parents in the management of symptoms and behavioral issues. Furthermore, the present study highlights the need of caring for parents of children with ASDs. 

## Figures and Tables

**Table 1:** Sample characteristics.

Children		
	No Insomnia	Insomnia
Mean (SD) age	8.62 (3.88)	7.29 (3.47)
**Gender**	N (%)	N (%)
Male	63 (63.6)	22 (22.2)
Female	10 (10.1)	4 (4.1)
Total n (%)	73 (73.7)	26 (26.3)
**Parents**		
Mean (SD) age	41 (6.2)	
**Gender**	N (%)	
Male	11 (11.1)	
Female	88 (88.9)	
**Professions**		
Employee	37 (37.4)	
Freelance	11 (11.1)	
Pensioned/Unemployed	39 (39.4)	
Other	12 (12.1)	

**Table 2:** Item–total correlation of the SDSC.

Item	Scale Mean If Item Deleted	Scale Variance If Item Deleted	Corrected Item-Total Correlation	Squared Multiple Correlation	Cronbach’s Alpha If Item Deleted
1	45.60	143.951	0.510	0.600	0.844
2	45.56	142.854	0.484	0.567	0.844
3	45.80	146.097	0.328	0.496	0.851
4	45.53	138.044	0.634	0.772	0.838
5	45.95	134.904	0.652	0.724	0.837
6	46.20	141.180	0.543	0.568	0.842
7	46.71	152.853	0.180	0.449	0.855
8	47.09	152.189	0.382	0.650	0.849
9	46.68	151.595	0.248	0.550	0.852
10	46.21	143.707	0.506	0.582	0.844
11	45.71	140.832	0.501	0.558	0.844
12	45.52	137.982	0.594	0.516	0.840
13	46.78	149.671	0.362	0.486	0.849
14	47.12	155.714	0.246	0.405	0.852
15	46.42	156.413	0.082	0.281	0.856
16	46.55	152.542	0.226	0.578	0.853
17	46.89	150.018	0.359	0.493	0.849
18	46.81	152.215	0.307	0.503	0.850
19	46.42	148.830	0.370	0.428	0.848
20	47.05	151.945	0.546	0.614	0.847
21	46.97	151.905	0.432	0.731	0.848
22	46.38	149.509	0.306	0.414	0.851
23	46.33	144.515	0.484	0.553	0.844
24	47.01	152.427	0.340	0.525	0.849
25	46.67	148.848	0.407	0.484	0.847
26	47.00	155.792	0.207	0.415	0.852

**Table 3:** Test–retest reliability of the SDSC.

SDSC Subscales	Test Mean (SD)	Retest Mean (SD)	ICC	Lower Bund	Upper Bund
DIMS	16.00 (7.27)	16.67 (5.74)	0.927	0.783	0.976
SBD	5.07 (2.15)	5.53 (2.80)	0.940	0.822	0.980
DA	3.53 (1.64)	3.53 (1.06)	0.849	0.551	0.949
SWTD	10.67 (3.28)	10.53 (3.35)	0.905	0.716	0.968
DOES	8.47 (3.68)	9.00 (4.44)	0.936	0.810	0.979
SHY	3.53 (1.45)	4.13 (1.50)	0.940	0.822	0.980
Total	47.27 (15.65)	49.40 (15.04)	0.972	0.916	0.991

DIMS = disorders initiating and maintaining sleep (7 items); SBD = sleep breathing disorders (3 items); DA = disorders of arousal (3 items); SWTD = sleep–wake transition disorders (6 items); DOES = disorders of excessive somnolence (5 items); SHY = sleep hyperhidrosis (2 items).

**Table 4:** Correlation between SDSC and PSQI.

PSQI Component	SDSC						
	DIMS	SBD	DA	SWTD	DOES	SHY	Total
1	0.741 **	0.198 *	0.378 **	0.515 **	0.442 **	0.117	0.714 **
2	0.695 **	−0.007	0.260 **	0.265 **	0.404 **	0.095	0.566 **
3	0.556 **	0.006	0.221 *	0.375 **	0.234 *	0.100	0.486 **
4	0.405 **	0.218 *	0.305 **	0.330 **	0.226 *	0.069	0.426 **
5	0.487 **	0.265 **	0.374 **	0.289 **	0.284 **	0.237 *	0.507 **
6	0.201 *	−0.103	0.137	0.313 **	0.251 *	−0.020	0.251 *
7	0.093	0.171	0.148	0.184	0.349 **	0.134	0.241 *
Total	0.752 **	0.131	0.415 **	0.544 **	0.518 **	0.143	0.745 **

DIMS = disorders initiating and maintaining sleep (7 items); SBD = sleep breathing disorders (3 items); DA = disorders of arousal (3 items); SWTD = sleep–wake transition disorders (6 items); DOES = disorders of excessive somnolence (5 items); SHY = sleep hyperhidrosis (2 items). * *p* < 0.05; ** *p* < 0.01.

**Table 5:** Correlation between SDSC and sleep disorders of parents as measured with GSDS.

SDSC Subscales	Subscales of the General Sleep Disturbance Scale					
	1	2	3	4	5	6
DIMS	0.328 **	0.177	0.407 **	0.438 **	0.303 **	0.276 **
SBD	−0.004	0.004	0.004	0.111	0.190	0.015
DA	0.263 **	0.031	0.320 **	0.351 **	0.297 **	0.247 *
SWTD	0.289 **	0.147	0.365 **	0.413 **	0.302 **	0.237 *
DOES	0.325 **	0.189	0.373 **	0.338 **	0.162	0.267 **
SHY	0.131	0.055	0.139	0.082	−0.002	0.042
Total	0.375 **	0.189	0.457 **	0.487 **	0.336 **	0.307 **

DIMS = disorders initiating and maintaining sleep (7 items); SBD = sleep breathing disorders (3 items); DA = disorders of arousal (3 items); SWTD = sleep–wake transition disorders (6 items); DOES = disorders of excessive somnolence (5 items); SHY = sleep hyperhidrosis (2 items). * *p* < 0.05; ** *p* < 0.01.

## Data Availability

Not applicable.

## References

[1] Thorpy M. (2017). International Classification of Sleep Disorders. Sleep Disorders Medicine: Basic Science, Technical Considerations and Clinical Aspects.

[2] Hirshkowitz M., Whiton K., Albert S.M., Alessi C., Bruni O., DonCarlos L., Hazen N., Herman J., Adams Hillard P.J., Katz E.S. (2015). National Sleep Foundation’s updated sleep duration recommendations: Final report. Sleep Health.

[3] Ohayon M.M., Smirne S. (2002). Prevalence and consequences of insomnia disorders in the general population of Italy. Sleep Med..

[4] Ohayon M.M. (2002). Epidemiology of insomnia: What we know and what we still need to learn. Sleep Med. Rev..

[5] Robinson-Shelton A., Malow B.A. (2015). Sleep Disturbances in Neurodevelopmental Disorders. Curr. Psychiatry Rep..

[6] Archbold K.H., Pituch K.J., Panahi P., Chervin R.D. (2002). Symptoms of sleep disturbances among children at two general pediatric clinics. J. Pediatr..

[7] American Psychiatric Association (2013). Diagnostic and Statistical Manual of Mental Disorders (DSM-5 (R)).

[8] Sen T., Spruyt K. (2020). Pediatric Sleep Tools: An Updated Literature Review. Front. Psychiatry.

[9] Markovich A.N., Gendron M.A., Corkum P.V. (2015). Validating the Children’s Sleep Habits Questionnaire Against Polysomnography and Actigraphy in School-Aged Children. Front. Psychiatry.

[10] Panzarella V., Giuliana G., Spinuzza P., La Mantia G., Maniscalco L., Pizzo G., Matranga D. (2021). Paediatric Sleep Questionnaire for Obstructive Sleep Apnoea Syndrome Screening: Is Sleep Quality Worthy of Note?. Appl. Sci..

[11] Bruni O., Ottaviano S., Guidetti V., Romoli M., Innocenzi M., Cortesi F., Giannotti F. (1996). The Sleep Disturbance Scale for Children (SDSC) Construct ion and validation of an instrument to evaluate sleep disturbances in childhood and adolescence. J. Sleep Res..

[12] Richdale A.L., Schreck K.A. (2009). Sleep problems in autism spectrum disorders: Prevalence, nature, & possible biopsychosocial aetiologies. Sleep Med. Rev..

[13] Devnani P.A., Hegde A.U. (2015). Autism and sleep disorders. J. Pediatr. Neurosci..

[14] Polimeni M.A., Richdale A.L., Francis A.J.P. (2005). A survey of sleep problems in autism, Asperger’s disorder and typically developing children. J. Intellect. Disabil. Res..

[15] Malow B.A., Marzec M.L., McGrew S.G., Wang L., Henderson L.M., Stone W.L. (2006). Characterizing Sleep in Children with Autism Spectrum Disorders: A Multidimensional Approach. Sleep.

[16] Mannion A., Leader G. (2013). Sleep Problems in Autism Spectrum Disorder: A Literature Review. Rev. J. Autism Dev. Disord..

[17] Blackmer A.B., Feinstein J.A. (2016). Management of Sleep Disorders in Children with Neurodevelopmental Disorders: A Review. Pharmacother. J. Hum. Pharmacol. Drug Ther..

[18] Mazurek M.O., Petroski G.F. (2014). Sleep problems in children with autism spectrum disorder: Examining the contributions of sensory over-responsivity and anxiety. Sleep Med..

[19] Goldman S.E., McGrew S., Johnson K.P., Richdale A.L., Clemons T., Malow B.A. (2011). Sleep is associated with problem behaviors in children and adolescents with Autism Spectrum Disorders. Res. Autism Spectr. Disord..

[20] Schreck A.K. (2004). Sleep problems as possible predictors of intensified symptoms of autism. Res. Dev. Disabil..

[21] Chen H., Yang T., Chen J., Chen L., Dai Y., Zhang J., Li L., Jia F., Wu L., Hao Y. (2021). Sleep Problems in Chil-dren with Autism Spectrum Disorder: A Multicenter Survey. BMC Psychiatry.

[22] Berardi A., Regoli E., Tofani M., Valente D., Fabbrini G., Fabbrini A., Ruggieri M., Panuccio F., Galeoto G. (2020). Tools to assess the quality of life in patients with Parkinson’s disease: A systematic review. Expert Rev. Pharm. Outcomes Res..

[23] Ioncoli M., Berardi A., Tofani M., Panuccio F., Servadio A., Valente D., Galeoto G. (2020). Crosscultural Validation of the Community Integration Questionnaire—Revised in an Italian Population. Occup. Ther. Int..

[24] Berardi A., Panuccio F., Pilli L., Tofani M., Valente D., Galeoto G. (2021). Evaluation instruments for executive functions in children and adolescents: A systematic review. Expert Rev. Pharmacoecon. Outcomes Res..

[25] Cavalli G., Galeoto G., Sogos C., Berardi A., Tofani M. (2021). The efficacy of executive function interventions in children with autism spectrum disorder: A systematic review and meta-analysis. Expert Rev. Neurother..

[26] Mammarella V., Arigliani E., Giovannone F., Cavalli G., Tofani M., Sogos C. (2022). Is It Hyperlexia? Toward a Deeper Understanding of Precocious Reading Skills in Two Cases of Children with Autism Spectrum Disorder. Clin. Ter..

[27] Tofani M., Galeoto G., Cazzetta D., Berardi A., Sansoni J., Valente D. (2019). Validation of the Pediatric Evaluation of Disability Inventory in an Italian Population with Autism Spectrum Disorder: A Cross-Sectional Study. Clin Ter..

[28] Tofani M., Giulia G.B., Lucibello L., Sabbadini M., Berardi A., Galeoto G., Field D., Castelli E. (2021). Seated postural control measure: Italian translation and validation in children with cerebral palsy. Prosthetics Orthot. Int..

[29] Tofani M., Castelli E., Sabbadini M., Berardi A., Murgia M., Servadio A., Galeoto G. (2020). Examining Reliability and Validity of the Jebsen-Taylor Hand Function Test Among Children with Cerebral Palsy. Percept. Mot. Ski..

[30] Cortese S., Wang F., Angriman M., Masi G., Bruni O. (2020). Sleep Disorders in Children and Adolescents with Autism Spectrum Disorder: Diagnosis, Epidemiology, and Management. CNS Drugs.

[31] Curcio G.G., Tempesta D., Scarlata S., Marzano C., Moroni F., Rossini P.M., Ferrara M., De Gennaro L. (2012). Validity of the Italian Version of the Pittsburgh Sleep Quality Index (PSQI). Neurol. Sci..

[32] Scialpi A., Mignolli E., De Vito C., Berardi A., Tofani M., Valente D., Galeoto G. (2022). Italian Validation of the Pittsburgh Sleep Quality Index (PSQI) in a Population of Healthy Children: A Cross Sectional Study. Int. J. Environ. Res. Public Health..

[33] Galeoto G., Scialpi A., Grassi M.L., Berardi A., Valente D., Tofani M., Paoloni M. (2019). General Sleep Disturbance Scale: Translation, cultural adaptation, and psychometric properties of the Italian version. CRANIO.

[34] Tang W., Cui Y., Babenko O. (2014). Internal Consistency: Do We Really Know What It Is and How to Assess It?. J. Psychol. Behav. Sci..

[35] Monticone M., Galeoto G., Berardi A., Tofani M. (2021). Psychometric Properties of Assessment Tools. Measuring Spinal Cord Injury.

[36] McLean K.J., Eack S.M., Bishop L. (2021). The impact of sleep quality on quality of life for autistic adults. Res. Autism Spectr. Disord..

[37] Al-Farsi O.A., Al-Farsi Y.M., Al-Sharbati M.M., Al-Adawi S. (2020). Sleep Problems among Parents of Children with Autism Spectrum Disorders in Oman: A Case Control Study. Kuwait Med. J..

[38] Deliens G., Leproult R., Schmitz R., Destrebecqz A., Peigneux P. (2015). Sleep Disturbances in Autism Spectrum Disorders. Rev. J. Autism Dev. Disord..

[39] Johnson C.R., Smith T., DeMand A., Lecavalier L., Evans V., Gurka M., Swiezy N., Bearss K., Scahill L. (2018). Exploring sleep quality of young children with autism spectrum disorder and disruptive behaviors. Sleep Med..

[40] Moore M., Evans V., Hanvey G., Johnson C. (2017). Assessment of Sleep in Children with Autism Spectrum Disorder. Children.

[41] Sadeh A. (2007). Consequences of Sleep Loss or Sleep Disruption in Children. Sleep Med. Clin..

[42] Gruber R., Wiebe S., Montecalvo L., Brunetti B., Amsel R., Carrier J. (2011). Impact of Sleep Restriction on Neurobehavioral Functioning of Children with Attention Deficit Hyperactivity Disorder. Sleep.

[43] Putois B., Leslie W., Gustin M.P., Challamel M.-J., Raoux A., Guignard-Perret A., Weick D., Sauzeau J.-B., Herbillon V., Zourou F. (2017). The French Sleep Disturbance Scale for Children. Sleep Med..

[44] Ağca S., Görker I., Turan F.N., Öztürk L. (2021). Validity and reliability of the Turkish version of Sleep Disturbance Scale for Children. Sleep Med..

[45] Huang M.-M., Qian Z., Wang J., Vaughn M.G., Lee Y.-L., Dong G.-H. (2014). Validation of the Sleep Disturbance Scale for Children and prevalence of parent-reported sleep disorder symptoms in Chinese children. Sleep Med..

[46] Saffari M., Gholamrezaei A., Saneian H., Attari A., Bruni O. (2014). Linguistic validation of the Sleep Disturbance Scale for Children (SDSC) in Iranian children with Persian language. Sleep Med..

[47] Ferreira V.R., Carvalho L.B., Ruotolo F., de Morais J.F., Prado L.B., Prado G.F. (2009). Sleep Disturbance Scale for Children: Translation, cultural adaptation, and validation. Sleep Med..

[48] Lopez-Wagner M.C., Hoffman C.D., Sweeney D.P., Hodge D., Gilliam J.E. (2008). Sleep Problems of Parents of Typically Developing Children and Parents of Children With Autism. J. Genet. Psychol..

[49] Al-Farsi O.A., Al-Farsi Y.M., Al-Sharbati M.M., Al-Adawi S. (2016). Stress, anxiety, and depression among parents of children with autism spectrum disorder in Oman: A case–control study. Neuropsychiatry Dis. Treat..

[50] Soltanifar A., Akbarzadeh F., Moharreri F., Soltanifar A., Ebrahimi A., Mokhber N., Minoocherhr A., Shojut S., Naqvi A. (2014). Comparison of Parental Stress among Mothers and Fathers of Children with Autistic Spectrum Disor-der in Iran. Iran. J. Nurs. Midwifery Res..

[51] Meltzer L.J. (2007). Brief Report: Sleep in Parents of Children with Autism Spectrum Disorders. J. Pediatr. Psychol..

[52] Schreck K.A., Mulick J.A. (2000). Parental Report of Sleep Problems in Children with Autism. J. Autism Dev. Disord..

